# Efficacy of fluoride varnishes for preventing enamel demineralization after interproximal enamel reduction. Qualitative and quantitative evaluation

**DOI:** 10.1371/journal.pone.0176389

**Published:** 2017-04-21

**Authors:** Ascensión Vicente, Antonio José Ortiz Ruiz, Belén Manuela González Paz, José García López, Luis-Alberto Bravo-González

**Affiliations:** 1 Department of Orthodontics, Faculty of Medicine, University of Murcia, Hospital Morales Meseguer, 2ª planta, C/ Marqués de los Vélez s/n, Murcia, Spain; 2 Department of Integral Pediatric Dentistry, Faculty of Medicine, University of Murcia, Hospital Morales Meseguer, 2ª planta, C/ Marqués de los Vélez s/n, Murcia, Spain; Istituto Di Ricerche Farmacologiche Mario Negri, ITALY

## Abstract

**Objectives:**

To evaluate quantitatively and qualitatively the changes produced to enamel after interproximal reduction and subjected to demineralization cycles, after applying a fluoride varnish (Profluorid) and a fluoride varnish containing tricalcium phosphate modified by fumaric acid (Clinpro White).

**Materials and methods:**

138 interproximal dental surfaces were divided into six groups: 1) Intact enamel; 2) Intact enamel + demineralization cycles (DC); 3) Interproximal Reduction (IR); 4) IR + DC; 5) IR + Profluorid + DC; 6) IR + Clinpro White + DC. IR was performed with a 0.5 mm cylindrical diamond bur. The weight percentage of calcium (Ca), phosphorous (P) and fluoride (F) were quantified by energy-dispersive X-ray spectrometry (EDX). Samples were examined under scanning electron microscopy (SEM).

**Results:**

The weight percentage of Ca was significantly higher (p<0.05) in Groups 1, 2 and 5 than Groups 4 and 6. No significant differences were detected in the weight percentage of Ca between Group 3 and the other groups (p>0.05). The weight percentage of P was similar among all six groups (p>0.05). F was detected on 65% of Group 6 surfaces. SEM images of Groups 4 and 6 showed signs of demineralization, while Group 5 did not.

**Conclusions:**

Profluorid application acts as a barrier against the demineralization of interproximally reduced enamel.

## Introduction

The interproximal reduction of dental enamel involves decreasing the enamel’s mesio-distal dimension through abrasion of its interproximal surfaces [1]. Its main objective is to create extra space to address dental overcrowding [2].

This procedure produces roughened enamel surfaces [3,4,5], which are susceptible to accumulating bacterial plaque. In addition, the reduced enamel is more vulnerable to demineralization [6]. In this context, any product that might prevent mineral loss and promote remineralization is worth investigating. But little research has looked into the use of remineralizing agents following interproximal reduction procedures and the results remain controversial.

Bonetti *et al*. [7] found that the topical application of GC Tooth Mousse containing casein phosphopeptide-amorphous calcium phosphate (CPP-ACP) was effective in promoting *in vitro* enamel remineralization after enamel reduction. This protective effect was also produced by toothpaste containing zinc-carbonate hydroxyapatite (Zn-CHA) [8]. But Paganelli *et al*. [9] found that *in vivo* application of MI Varnish containing casein phosphopeptide-amorphous calcium phosphate and fluoride (CPP-ACPF) to teeth that had undergone enamel reduction did not have a greater protective effect than that of the patient’s own saliva. Jarjoura *et al*. [10] concluded that topical applications of fluoride in patients who had undergone enamel reduction and then been exposed to fluoridated water and toothpastes did not produce any additional benefit.

Fluoride varnishes adhere to dental surfaces, resulting in longer contact with enamel than toothpastes and creams. In addition to their remineralizing action resulting from ion release, it is likely that these products act as a physical barrier that protects the enamel against acid attack [11]. Although the use of varnishes to prevent white spots during orthodontic treatment has been widely investigated [12,13,14,15], their effects following interproximal reduction procedures–as far as we are aware–have been studied just by Peng et al. [16]. They measured microhardness, density and mineral loss after using a fluoride varnish and resin infiltration on stripped enamel surfaces [16]. To the best of our knowledge, the calcium, phosphorous and fluoride F fluoride content of stripped enamel surfaces after using a fluoride varnish has not been studied.

So the objectives of the present study were: to determine by means of energy dispersive X-ray spectrometry (EDX) the calcium (Ca), phosphorous (P) and fluoride (F) content of enamel after interproximal reduction subjected to cycles of demineralization, after the application of a fluoride varnish (Profluorid) and a fluoride varnish containing tricalcium phosphate modified by fumaric acid (Clinpro White), and to evaluate by means of scanning electron microscopy (SEM), the morphological changes produced in the enamel.

## Materials and methods

### Sample preparation

The study design was approved by the University of Murcia bioethics committee. Written informed consent was obtained from all participants. The sample consisted of 69 lower permanent incisors extracted for orthodontic or periodontal reasons. Any teeth that presented interproximal and/or cervical caries, restorations, or excessive wear were excluded. The teeth were stored in distilled water for up to one month.

After setting the teeth in plasticine (Art. 70 JOVI, S.A, Barcelona, Spain) to ensure that the points of interdental contact were correct, 15 sets of four teeth, one set of two teeth, and one set of seven were set in type IV dental plaster.

### Interproximal enamel reduction procedure

Enamel reduction was carried out in the area of the point of contact between adjacent teeth. In both the 15 sets of four incisors and the single set of two incisors, interproximal enamel reduction was performed on the mesial and distal surfaces of each tooth, with the exception of the distal surfaces at the ends of the sets. The set of seven teeth did not undergo reduction.

Interproximal enamel reduction was performed with a 0.5 mm. cylindrical diamond bur (F.G 8392–016 fine grain 30 mm Komet Dental, Gebr. Brasseler GmbH & Co. Lemgo. Germany) under water-cooling. During the procedure, adjacent teeth were protected by metal matrix bands (Hawe Steel Matrix Bands, Kerr. Japan). The depth of the enamel reduction was checked with dental measuring devices (IPR-Distance Control. Intensiv SA. Montagnola, Switzerland) being the reference for measurements the adjacent tooth to which the interproximal reduction was done. A 0.5 mm gauge was used when the adjacent surface was intact and a 1 mm gauge when the adjacent tooth had already undergone reduction. A new bur was fitted after every 20 reduction procedures. The same clinician performed all procedures.

### Experimental groups

Teeth in all sets were sectioned along their main axis using a diamond disc (Komet Dental, Gebr. Brasseler GmbH & Co. Lemgo. Germany), separating the mesial and distal surfaces. All samples were also sectioned horizontally at the cementoenamel junction, obtaining 138 proximal dental surfaces, 92 with interproximal enamel reduction and 46 with the enamel intact.

The 138 dental surfaces were divided into six groups (n = 23): 1) Intact enamel; 2) Intact enamel + demineralization cycles (DC); 3) interproximal enamel reduction (IR); 4) IR + DC; 5) IR + Profluorid + DC; 6) IR + Clinpro White + DC.

### Varnish application

Profluorid^®^ Varnish (VOCO GmbH. Cuxhaven, Germany) and Clinpro White Varnish (3M ESPE, St. Paul, MN, USA) were applied to the interproximal dental surfaces in Groups 5 and 6 respectively. Table 1 details the composition of these products. They were applied to the dental surfaces, which had been cleaned and dried previously, following the manufacturers’ instructions. The varnishes were left to dry for a minute before being placed in artificial saliva.

**Table 1:** Products composition according material safety data sheets (MSDS).

Varnish	Composition	% by Wt
VOCO Profluorid varnish	Ethanol	10–25
	Sodium fluoride	
		
Clinpro White varnish with TCP	Pentaerythritol glycerol ester of	30–75*
	colophony resin	10–15*
	N-Hexane	1–5*
	Ethyl alcohol	1–5*
	Sodium fluoride	1–5*
	Flavor enhancer	1–5*
	Thickener	1–5*
	Food grade flavor	1–5*
	Modified tricalcium phosphate	<5*

*The specific chemical identity and/or exact percentage (concentration) of this compositon has been withheld as a trade secret.

### Sample storage and demineralization cycles

Groups 1 and 3 were kept in artificial saliva at 37° for 8 days. The saliva composition used as storage medium was: 1% carmellose sodium, 13% sorbitol, 0.12% potassium chloride, 0.084% sodium chloride, 0.005% magnesium chloride hexahydrate, 0.015% calcium chloride anhydrous, 0.017% potassium phosphate dibasic, and 0.1% Nigapin® sodium. The saliva pH was adjusted and maintained at 6.57. [17]

Group 2, 4, 5 and 6 samples were submerged in artificial saliva at 37°C for 8 days, and subjected to demineralization cycles as follows: samples were placed in a demineralizing solution for 2 hours, three times per day, and returned to artificial saliva between the 2-hour cycles. Samples were washed in distilled water at each change of medium. The demineralization solution and the artificial saliva were changed every 48 hours.

The composition of the demineralization solution was as follows: 2.2 mM calcium chloride (CaCl_2_ 2H_2_O); 2.2 mM monosodium phosphate (NaH_2_PO_4 7_H_2_O), 0.05 mM lactic acid; pH was adjusted to 4.5 with 50% sodium hydroxide (NaOH). [18]

### Sample preparation for EDX/SEM analysis

Tooth surfaces in all groups were washed in distilled water and Group 5 and 6 samples were cleaned with a dental prophylaxis brush to eliminate the varnish to allow quantification of the enamel elements, and to observe enamel morphology. Then, all surfaces were given an ultrasonic bath for 60 minutes at room temperature, in order to eliminate any remaining varnish from the surfaces, or any other impurity that might interfere with EDX and SEM observation.

#### EDX analysis

Twenty surfaces in each group were coated with carbon and analyzed using a JEOL-6100 scanning electron microscope (Jeol Ltd., Tokyo, Japan) equipped with an INCA energy dispersive X-ray microanalysis system (Oxford Instruments Analytical, Oxfordshire, United Kingdom) at 20 KV, with a counting time of 100 s per source.

Sample size was calculated using the program “G*Power 3.1.9.2 for Mac” [19]. The statistical power was calculated for a one way ANOVA test considering an alpha error probability of 0.05. The resulting power for n = 120 at an alpha level of 0.05, was 0.88

The elements quantified were: Ca (weight %), P (weight %) and F (weight %). Using the Ca and P values obtained, Ca/P stoichiometric ratios were calculated using the following formula: Ca (mol) /P (mol) % = [Ca (weight %) /40.08 (g/mol)]/ [P (weight %)/30.97 (g/mol)], the molecular masses of Ca and P being 40.08 and 30.97 respectively.

#### SEM analysis

Three samples from each group were coated with gold and examined under x1000 magnification at 20 KV. The most representative images were captured and stored.

### Statistical analysis

Statistical analysis was performed using the SPSS 19.0 statistical software package (IBM SPSS Inc., New York, USA).

Weight percentage values for Ca and P, as well as Ca/P stoichiometric ratios underwent the Kolmogorov-Smirnov normal distribution test (p<0.05) and the Levene test for homogeneity of variance (p<0.05).

Data corresponding to weight percentages of Ca and Ca/P stoichiometric ratios fulfilled normality criteria (p>0.05) and homogeneity of variance (p>0.05), and so were analyzed with one-factor ANOVA (p<0.05) and the least significant difference test (p<0.05) in the event ANOVA was significant, according to recommendations [20].

Data corresponding to weight percentages of P did not fulfill normality criteria (p>0.05) or homogeneity of variance (p>0.05), and so were analyzed using the Kruskal-Wallis test (p<0.05).

## Results

### EDX

One-factor ANOVA identified statistically significant differences (p = 0.00) in the weight percentage of Ca between different groups. The least significant difference test found that the weight percentage of Ca was significantly greater in the Intact enamel, Intact enamel + DC, and IR + Profluorid + DC groups than in the IR + DC (p = 0.00; p = 0.01; and p = 0.02 respectively) and IR + Clinpro + DC groups (p = 0.00; p = 0.01; and p = 0.02 respectively). No significant differences were found (p>0.05) in other comparisons between groups, drawing attention to the fact that the IR group did not show differences in the weight percentage of Ca on sample surfaces in comparison with the rest of the groups (Table 2).

**Table 2:** Weight percentage (mean±standard deviation) of calcium (Ca), phosphorous (P) and fluoride (F). Ratio Ca/P (mol/mol).

Groups	Ca	P	Ca/P	F
**Intact enamel**	40.62±2.26 **A**	18.40±0.59	1.70±0.10	0
**Intact enamel + DC**	40.25±2.14 **A**	18.54±0.47	1.68±0.08	0
**IR**	39.37±2.57	18.19±0.55	1.67±0.09	0
**IR+DC**	38.59±1.64 **B**	18.01±0.60	1.66±0.05	0
**IR + Profluorid + DC**	40.10±1.98 **A**	18.38±0.47	1.68±0.05	0
**IR + Clinpro + DC**	38.62±1.40 **B**	18. 01±0.86	1.66±0.08	0.71±0.30

IR: interproximal reduction, DC: demineralization cycles.

For each column, different uppercase letters indicate significant differences (p<0.05). Groups without a letter did not show significant differences with any other (p>0.05).

For Ca/P stoichiometric ratio data, one-factor ANOVA did not identify significant differences between groups (p = 0.37) (Table 1).

As for P weight percentage values, the Kruskal-Wallis test did not detect significant differences (p = 0,06) between the six groups (Table 2).

On 65% of reduced enamel surfaces treated with Clinpro White varnish, the presence of F was detected (Table 2).

### SEM

**Fig 1:**
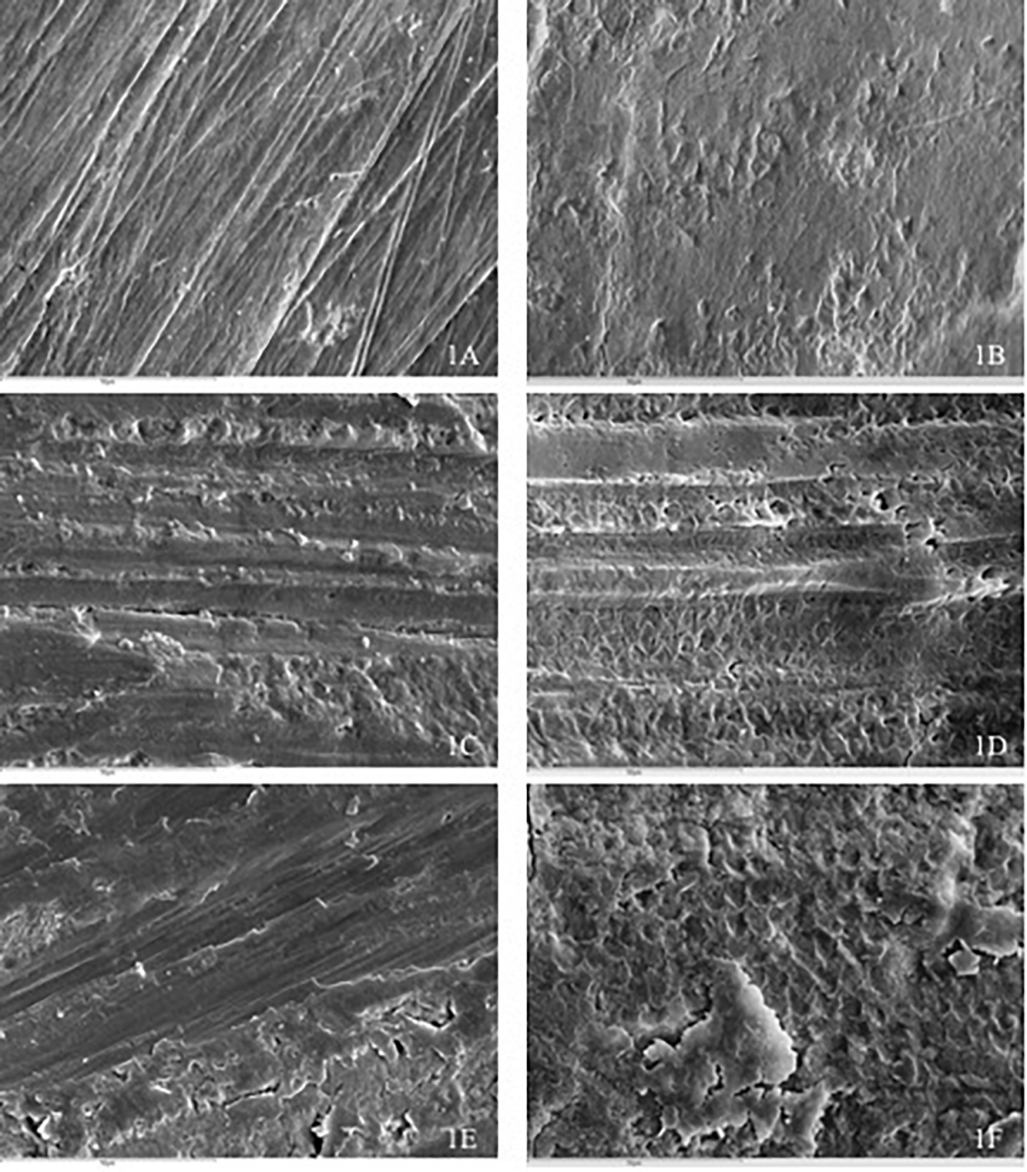
SEM micrograph (x1000). **A)** Intact enamel group; **B)** Intact enamel + DC; **C)** IR; **D)** IR + DC; **E)** IR + Profluorid + DC; **F)** IR + Clinpro + DC.

SEM images of intact enamel without demineralization showed typical enamel surface layers, with small areas of erosion and wear (Fig 1A). For Intact enamel + DC, images showed a process of early demineralization, with areas presenting slight dissolution of interprismatic tissue, without affectation of the nuclei of the prisms (Fig 1B). Fig 1C shows an image of an Enamel IR group sample, in which grooves and irregularities can be seen caused by the bur used for enamel reduction. On the dental surfaces of IR + DC group samples, areas with preferential destruction of the centre of the prism nuclei and indiscriminate destruction could be observed in some areas (Fig 1D). RI + Profluorid +CD images did not show demineralization, and the images were similar to the IR group (Fig 1E). But in IR + Clinpro + DC sample images, a typical honeycomb pattern of demineralization could be seen, with preferential dissolution of the prism nuclei; in spite of sample cleaning with a dental prophylaxis brush, remains of the varnish were observed in most of the images (Fig 1F).

## Discussion

The objective of this study was to value quantitatively and qualitatively the changes to dental enamel that had undergone interproximal reduction after application or non-application of Profluorid and Clinpro varnishes, after subjecting samples to demineralization cycles.

The study used human lower incisors. As the teeth came from different individuals, an initial set-up was made with plasticine in order to ensure that interdental contact points were correct and to determine the interproximal enamel reduction performed. As in other studies of enamel reduction that have evaluated the use of remineralization agents, the quantity of enamel removed from each interproximal surface was of 0.5 mm [7, 8,10]. Reduction was performed using safe-tipped burs at high speed, as this is one of the most widely used and documented techniques in the scientific literature [21,22]. Final polishing was not carried out, as this does not appear to influence the degree of enamel demineralization [6].

In order to simulate the oral medium, samples were subjected to daily demineralization cycles, remaining in artificial saliva the rest of the time. The cycles were spaced 2 hours apart to ensure complete pH recovery. Oral pH recovery time after ingesting food would appear to range between 30 and 50 minutes [23]; but recovery time may be longer in interproximal areas due to the reduced saliva access to these areas [24].

To distinguish between Ca and P loss as a consequence of demineralization and as a consequence of interproximal reduction, the study included two control groups, one with intact enamel and another with enamel reduction but without demineralization cycles.

The main components of hydroxyapatite are Ca and P, and so these elements were the objects of study [25]. The Ca/P ratio was also determined as this is an indicator of dental tissue mineralization [9].

The results showed that Ca and P content of enamel that had undergone reduction was slightly less than intact enamel. This confirms the results of other researchers who have found that mineral density [25, 26], as well as the weight percentage of Ca and P decrease from the upper surface layers to the more internal layers [25].

Ca values were lower in the intact enamel and the IR groups subjected to demineralization cycles, than in the corresponding non-demineralized groups, but without statistically significant differences. So while demineralization cycles were seen to produce demineralization, this was not sharp. But Ca values obtained in the IR + DC group, were significantly lower than in the Intact enamel + DC; SEM images also showed greater demineralization in the IR+DC group than the Intact enamel + DC group. This indicates that the surface with enamel reduction, being a less mineralized surface than intact enamel, was more susceptible to demineralization, a finding that concurs with other studies [6,7].

Samples that underwent interproximal reduction without demineralization cycles (IR group) produced lower surface Ca values than the intact enamel groups, regardless of whether they underwent demineralization cycles or not, and lower values than the IR+ Profluorid + DC, although differences were not significant. In order to induce demineralization phenomena on enamel dental surfaces that are not adequately protected, decreases in pH would appear to be a necessity. When pH was kept at around 6.57, no signs of demineralization were seen on enamel that had undergone interproximal reduction.

Results obtained with Profluorid showed that its application after enamel reduction protected the dental surface against demineralization, as the weight percentage of Ca was significantly higher in this group than in IR + DC group; SEM images in the IR + Profluorid + DC group, showed enamel that had not suffered demineralization, presenting a similar appearance to images in the IR group. But Clin Pro White application was less effective than Profluorid, as the weight percentage of Ca in the Clinpro White Varnish group was similar to the IR + DC and significantly lower than in the IR + Profluorid + DC group; SEM images confirmed that enamel had undergone a degree of demineralization similar to the IR + DC group.

Clinpro White Varnish is a fluoride varnish containing tricalcium phosphate modified by fumaric acid (fTCP). Shen *et al*. [27] observed that Profluorid, which does not include calcium ions in its composition, releases calcium ions, this release being similar to Clinpro White Varnish. Clinpro White Varnish provides low calcium and inorganic phosphate ion release that may be explained by the small quantity of fTCP added to the varnish or by the low solubility of tricalcium phosphate [28].

As for F release, although both varnishes have similar content, F was not detected in any of the samples treated with Profluorid, while F was found on 65% of the surfaces treated with Clinpro White Varnish. While some authors have observed that after 24 hours Profluorid has released approximately 17% of its fluoride content (201.34 μmol/g) and Clinpro White Varnish has released 16% (175 .87 μmol/g) [27], others have found that after 24 hours the percentage of fluoride released by Clinpro White Varnish was 6% and 20% after 7 days [28]. Bolis *et al*. [29] observed that Clinpro White Varnish and Proflluorid showed similar ranges of fluoride release and fluoride capture by the enamel surfaces. These differences in results and observations could be due to the different storage media used in different studies, as it has been seen that the storage medium influences the amount and rate of fluoride release from fluoridated restorative materials [30].

In the present study, the weight percentage of P was similar between the all the groups evaluated. This finding coincides with other research in which P also remained stable [31].

Perhaps the present findings can be explained by the different viscosity of the varnishes evaluated. In agreement with the present study, Shen *et al*. [27] observed that Clinpro White Varnish was very viscous and a greater quantity of varnish was needed to cover the same surface than Profluorid. So the greater fluidity of Profluorid, could favor both more adhesive contact with the enamel and the formation of a more even and homogenous layer of varnish. It could be that the lesser wettability of Clinpro White Varnish did not allow adequate diffusion of the varnish over the enamel surface, producing less contact between the varnish and the dental tissue and a more irregular varnish layer, allowing the diffusion of acids and the demineralization of the enamel beneath. Although F was detected in some Clinpro White Varnish samples, SEM images showed signs of demineralization; so it is not certain that the fluoride detected was fluoride captured by the enamel. Fluoride catalyzes the diffusion of Ca and phosphate over the dental surface, which favors remineralization of the crystalline structures in dental cavities to produce fluorapatite crystals, which is the most resistant crystalline phase [32]. The Ca/F atom mol ratio of fluorapatite is 5 [33]. In the present study the fluoride values obtained were very low and so the Ca/F ratio would be well above 5. This means that the fluoride detected would not form part of the fluorapatite, so it might be that other more soluble fluorine compounds had formed, or it could be–and we consider this possibility more likely–that this fluoride corresponds to the remains of varnish which were seen in some SEM images of teeth treated with Clinpro White Varnish, in spite of attempts to eliminate the varnish with a dental prophylaxis brush.

To date just one research into the use of fluoride varnishes after dental enamel reduction procedures has been published. Peng *et al*. state in their study that fluoride varnish and resin infiltration may provide an enamel protection from acid challenge. Both treatments enhanced the surface microhardness of the enamel after interproximal reduction [16]. Their results are in accordance with ours, particularly for the protective effect on enamel that we found in our research with Profluorid® varnish.

The use of remineralizing agents such as toothpastes [8,10] and tooth mousses [7,9] has been widely studied. But from the clinical point of view, the use of fluoride varnish could be more effective, given that after interproximal enamel reduction in cases of dental overcrowding, new interdental contact points are soon established, limiting toothpaste and dental cream access to the reduced interproximal surfaces. But a fluoride varnish will remain adhered to the surface, maintaining its contact with the enamel for longer than toothpaste or dental cream.

Of the two varnishes evaluated, Profluorid obtained better results and its greater fluidity makes it easier to handle when applying the product after interproximal enamel reduction.

*In vitro* studies necessarily present certain limitations. For this reason, the results need confirmation in *in vivo* studies that pay attention to those variables that cannot be reproduced in the laboratory.

## Conclusions

- The use of Profluorid after interproximal dental enamel reduction of incisors prevents the loss of calcium provoked by demineralization cycles. Clinpro White Varnish was not seen to have this effect. Phosphorous values were similar with both varnishes; fluoride was detected only in the group in which Clinpro White Varnish was applied.
- SEM images captured revealed the presence of demineralization on surfaces with interproximal reduction subjected to demineralization cycles and Clinpro White application, while surfaces protected by Profluorid showed no signs of demineralization.
  Profluorid application could act as a ‘barrier’ against the demineralization processes suffered by enamel following interproximal reduction.
